# β-catenin-mediated YAP signaling promotes human glioma growth

**DOI:** 10.1186/s13046-017-0606-1

**Published:** 2017-09-29

**Authors:** Yan Wang, Peng Pan, Zhaohao Wang, Yu Zhang, Peng Xie, Decheng Geng, Yang Jiang, Rutong Yu, Xiuping Zhou

**Affiliations:** 1 Insititute of Nervous System Diseases, Xuzhou Medical University, 84 West Huai-hai Road, Xuzhou, Jiangsu 221002 People’s Republic of China; 2 Brain Hospital, Affiliated Hospital of Xuzhou Medical University, Xuzhou, Jiangsu China; 3 The Graduate School, Xuzhou Medical University, Xuzhou, Jiangsu China; 4 Present address: Department of Neurosurgery, Xuzhou Cancer Hospital, Xuzhou, Jiangsu China; 5 Jiangsu Center for the Collaboration and Innovation of Cancer Biotherapy, Cancer Institute, Xuzhou Medical University, Xuzhou, Jiangsu China

**Keywords:** Glioma, Proliferation, YAP, β-catenin, GSK3β, Nude mice

## Abstract

**Background:**

Hippo/YAP pathway is known to be important for development, growth and organogenesis, and dysregulation of this pathway leads to tumor progression.We and others find that YAP is up-regulated in human gliomas and associated with worse prognosis of patients. However, the role and mechanism of YAP in glioma progression is largely unknown.

**Methods:**

The expression of YAP in glioma tissues was detected by quantitative polymerase chain reaction (qPCR) and immunoblotting. The effect of YAP on glioma progression was examined using cell growth assays and intracranial glioma model. The effect of YAP on β-catenin protein level, subcellular location and transcription activity was examined by immunoblotting, immunofluorescence and RT-PCR.

**Results:**

Firstly, knockdown of YAP inhibited glioma cell proliferation in vitro and tumor growth in vivo. In addition, YAP modulated the protein level, subcellular location and transcription activity of β-catenin via regulating the activity of GSK3β. Lastly, β-catenin partially mediated the effect of YAP on glioma cell proliferation.

**Conclusion:**

Our findings identify that YAP promotes human glioma growth through enhancing Wnt/β-catenin signaling. In addition, this study provides a new crosstalk mechanism between Hippo/YAP and Wnt/β-catenin pathways, which suggests a new strategy for human glioma treatment.

**Electronic supplementary material:**

The online version of this article (10.1186/s13046-017-0606-1) contains supplementary material, which is available to authorized users.

## Background

Malignant glioma is the most common brain cancer. Affected patients are usually treated with a combined approach of surgery, chemotherapy and radiation therapy, but the median survival time is only 12–15 months [1]. Therefore, understanding the molecular mechanism underlying pathogenesis of the disease is critical to identify specific molecular targets for glioma treatment.

Recent studies have identified the Hippo/YAP signaling as a key mechanism that controls organ size by imping on cell growth and proliferation [2, 3]. The MST1/2-WW45 complex phosphorylates and activates LATS1/2-MOB complex, which in turn phosphorylates oncogenic protein YAP and TAZ. YAP and TAZ normally function in the nucleus as a co-activator for the TEAD/TEF family transcription factors to promote cell growth, proliferation, and survival. Phosphorylation of YAP and TAZ promotes their interaction with 14–3-3, leading to their cytoplasmic retention. Thus, the Hippo pathway activation inhibits transcriptional activity of YAP and TAZ [2, 3]. Accumulating evidence suggests that the Hippo/YAP pathway is dysregulated in many human cancers. Elevated YAP/TAZ expression or nuclear enrichment has been observed in many types of cancers, including liver, breast, lung, colon, ovary and others [4–6]. Orr et al. found that YAP1 is up-regulated in the clinically aggressive glioblastoma subtypes (classical and mesenchymal) and is associated with the worst patient median survival [7]. Our pervious study also identified that YAP1/TAZ increased and meanwhile those of p-YAP1/p-TAZ and LATS1/2 decreased in gliomas. YAP1/TAZ-BIRC5 might be abnormally activated due to LATS1/2 down-regulation, which in turn promotes the occurrence and development of gliomas [8, 9]. Although YAP is up-regulated in gliomas and associated with worse prognosis of patients, the role and mechanism of YAP in gliomas is largely unknown.

The canonical Wnt pathway regulates many biological processes, including cell proliferation, cell fate decision, axis formation and organ development during embryonic development and tissue homeostasis [10, 11]. The key effector in this pathway is the transcriptional activator β-catenin. Without Wnt, cytoplasmic β-catenin is phosphorylated by GSK3β and then degraded. Upon Wnt stimulation, β-catenin is unphosphorylated, stabilized and then enters the nucleus to promote the transcription of downstream target genes, such as c-myc and cyclin D1. Dysregulation of Wnt/β-catenin pathway has been implicated in a number of cancers [12]. Patients with high grade astrocytoma have a higher expression level of β-catenin and knockdown of β-catenin in human glioma cells inhibits cell proliferation and induces apoptosis [13–15].

Several studies have shown that the Hippo/YAP pathway genetically and functionally interacts with Wnt/β-catenin signaling. For instance, Varelas et al. have shown that cytoplasmic TAZ binds to dishevelled and inhibits Wnt signaling [16]. Moreover, Heallen et al. reported that Hippo/YAP signaling inhibits Wnt/β-catenin pathway in developing heart [17]. Later, Imajo et al. found that YAP and TAZ interact with β-catenin to block its nuclear localization, inhibiting Wnt/β-catenin signaling [18]. However, Konsavage et al. reported that YAP is a direct Wnt/β-catenin target gene and its expression is required for colorectal carcinoma cell growth [19]. In addition, Azzolin and his colleagues identified a mechanism that Wnt/β-catenin signaling increases the level of YAP/TAZ [20]. Recently, Park et al. showed that the transcriptional regulators YAP/TAZ are found to be the key downstream effectors of alternative Wnt signaling, as well as negative regulation of canonical Wnt/β-catenin signaling [21]. These reports suggest that the Hippo/YAP pathway and Wnt/β-catenin signaling could regulate each other through multiple mechanisms, depending on biological contexts. Since dysregulation of Hippo/YAP and Wnt/β-catenin pathways leads to tumor progression, elucidating the mutual regulatory mechanism of these two pathways might reveal potential targets for tumor therapeutic intervention.

Whether and how YAP regulates β-catenin to promote glioma cell proliferation remains poorly understood. In this study, we demonstrated that knockdown of YAP expression inhibited glioma cell proliferation in vitro and tumor growth in vivo. YAP modulated the protein level, subcellular location and transcription activity of β-catenin via regulating the activity of GSK3β. At last, β-catenin mediated the effect of YAP on glioma cell proliferation. These results provide a novel mechanism that YAP promotes human glioma cell growth via β-catenin activation.

## Methods

### Glioma and nontumor samples

A total of 26 human glioma samples and 13 nontumor brain tissues (decompressive surgery) were obtained from Affiliated Hospital of Xuzhou Medical University. All of the glioma samples used in this study were astrocytomas, which were histologically diagnosed according to the World Health Organization grading system (12 of WHO grade II, 5 of WHO grade III, 3 of WHO grade III-IV, 6 of WHO grade IV). All the glioma and nontumor brain tissues had been collected immediately after surgical resection and stored in −80 °C. Written informed consent was obtained from the patients and the study was approved by the Ethic Committee of the hospital.

### Cell culture

The U87 and U251 glioma cells were purchased from Shanghai Cell Bank, Type Culture Collection Committee, Chinese Academy of Science. Cells were cultured in DMEM/F-12 (Gibco) media, supplemented with 10% fetal bovine serum (FBS, BioInd, Israel).

### Antibodies and plasmids

Antibodies against YAP1 were purchased from Abcam (Cambridge, MA). Antibodies specific for GSK3β, p-GSK3β, β-catenin, Non-phospho active β-catenin ^(Ser33/37/Thr41)^, p-β-catenin and GAPDH were obtained from Cell Signaling Technology (Beverly, MA). Antibody against Ki67 was purchased from Thermo. β-catenin^CA^ and β-catenin^WT^ plasmids were kindly donated by Prof. Zhen-Ge Luo at the Institute of Neuroscience and Key Laboratory of Neurobiology, Chinese Academy of Sciences. YAP wild type and YAP S94A plasmids were kindly gifted by Prof. Bin Zhao at the Life Sciences Institute of Zhejiang University.

### Establishment of YAP down-regulation or over-expression glioma cells

To establish YAP down-regulation glioma cells, short hairpin RNA (shRNA) targeting human YAP1 was inserted into lentiviral pLL3.7 backbone at HpaI and XhoI sites. For over-expression of YAP, the YAP2 cDNA was inserted into the pWPXLd backbone with GFP tag using PacI and MluI sites. The sequences (Sangon Biotech Shanghai) of shYAP were: F: 5′-TGCAGCAGAATATGATGAACTTCAAGAGAGTTCATCATATTCTGCTGCTTTTTTC-3′; R: 5′-TCGAGAAAAAAGCAGCAGAATATGATGAACTCTCTTGAAGTTCATCATATT CTGCTGCA-3′. Primers sequences for YAP2 were: F: 5′-CCTTAATTAAATGGATCCCGGGCAGCAG-3′; R: 5′-CGACGCGTCCCTATAACCATGTAAGAAAGC-3′.

### RNA extraction, cDNA synthesis and quantitative PCR (qPCR)

RNA was extracted from the human glioma cell lines U87 and U251 with YAP down-regulation or over-expression and the cDNA was synthesized using reverse transcription reagents (Roche, Basel, Switzerland) according to the manufacturer’s protocol [8]. Quantitative PCR was performed on an ABI 7500 qPCR instrument (Applied Biosystems, Carlsbad, CA, USA) using SYBR Green. Primers are as follows: YAP-F, 5′-CACAGCTCAGCATCTTCGAC-3′; YAP-R, 5′-TATTCTGCTGCACTGGTGGA-3′; β-catenin-F, 5′-CTTACACCCACCATCCCACT-3′; β-catenin-R, 5′-CCTCCACAAATTGCTGCTGT-3′; β-actin-F, 5′-CATGTACGTTGCTATCCAGGC-3′; β-actin-R,5′-CTCCTTAATGTCACGCACGAT-3′.

### Cellular fractionation and Immunoblotting

Cellular fractionation was conducted by using Membrane and Cytosol Protein Extraction Kit (Biovision), according to the instruction of manufacturer. Equal amount of protein lysates were subjected to 10% SDS-PAGE and then transferred to 0.45 μm pore size PVDF membrane (Millipore). After blocking with 5% non-fat milk, the membrane was probed with primary antibodies at 4 °C overnight and secondary antibodies at room temperature for 1 h. Bound antibodies were detected by the Pierce ECL Plus Western Blotting Substrate (Thermo Fisher, Waltham, MA, USA) and exposed to X-ray films. Band densities were quantified by ImageJ Software (Wayne Rasband, National Institutes of Health, MD). The relative amount of proteins was determined by normalizing the densitometry value of interest to that of the loading control [22].

### EdU incorporation assay

Cells were seeded into 96-well plates at 7 × 10^3^ cells per well. Twenty-four hours later, the cells were exposed to 50 μM of 5-ethynyl-20-deoxyuridine (EdU; Ribobio, Guangzhou, China) for additional 2 h at 37 °C. Then, the cells were fixed with 4% paraformaldehyde for 20 min and treated with 0.5% Triton-X-100 for another 20 min at room temperature. After being washed with PBS for five times, the cells were reacted with 100 μL of 1× Apollo® reaction cocktail for 30 min. Thereafter, the DNA contents of cells were stained with 100 μL of Hoechst 33,342 (5 μg/mL) for 20 min and visualized under a fluorescent microscope (IX71, Olympus, Tokyo, Japan). Data were obtained from three independent assays performed in triplicate [23].

### Cell counting Kit-8 assay

Cell growth curves were obtained by detecting the cell viability with the Cell Counting Kit-8 (CCK-8, Dojindo, Japan) assay every 24 h according to the manufacturer’s instruction.

### Colony formation assay

Three days after lentivirus infection, cells were seeded into 6-well plate (300 cells per well). The medium was changed at three days intervals. After 14 days of culture at 37 °C, the colonies were washed with PBS and fixed with 4% paraformaldehyde for 30 min at room temperature. The colonies were then stained with 0.05% crystal violet for 10 min, washed with water and air-dried. The total number of colonies with more than 50 cells was counted.

### Intracranial model of glioma in nude mice

Intracranial model of glioma in nude mice was performed according to our previous study [22]. All the in vivo experiments were carried out with ethical committee approval and met the standards required by the guidelines of Xuzhou Medical University. Female athymic nude mice aged 4–6 weeks and weighed 20 g were obtained from Experimental Animal Center of Xuzhou Medical University. Mice were randomly divided into two groups (*n* = 6 per group) and were anesthetized by intraperitoneal injection of 3% chloral hydrate (0.01 mL/body weight (g)). According to the coordinates, a small burr hole, 2 mm diameter, was drilled with 1.4 mm away from the midline at the right side of the cranium. The head of syringe was held in a horizontal position and shYAP or scramble U87 cells (1 × 10^6^) were injected into right striatum at a depth of 2.6 mm using a small animal stereotactic apparatus. The cells were injected slowly within 10 min at a rate of 0.5 μL/s and the syringe stayed for 10 min before withdrawing. The burr hole was sealed with bone wax, strict aseptic conditions were applied in the whole experiments.

To obtain the survival curve, the mice were sacrificed when they appeared hemiplegia, listlessness, cachexia and other neurological symptoms or without neurological symptoms at 50 days after transplantation. The cryosections of brain were subjected to H.E. staining and the tumor volume was calculated according to the formula V = 1/2 ab^2^ with ‘a’ representing the longest diameter and ‘b’ representing the shortest diameter.

### Immunofluorescence

Immunofluorescence was performed according to our previous study [22]. The brain sections containing the tumor or cells were incubated with 0.3% triton X-100 followed by 10% goat serum and then were exposed to a primary antibody (anti-β-catenin, anti-active-β-catenin and anti-Ki67) at 4 °C overnight. To visualize the positive cells, the sections or cells were incubated with Alexa fluor conjugated second antibody. The DAPI was used to stain cell nuclear. The sections were visualized under a fluorescent microscope (IX71 Olympus, Tokyo, Japan).

### Statistical analysis

Data were analyzed by using Student’s *t* test or ANOVA for multiple comparisons. In all analysis, quantitative data were obtained from at least three independent experiments and expressed as mean ± SEM. *P* values less than 0.05 were considered statistically significant (*P < 0.05, **P < 0.01, ***P < 0.001).

## Results

### YAP is highly expressed in gliomas and knockdown of YAP inhibits glioma cell proliferation in vitro

**Fig. 1:**
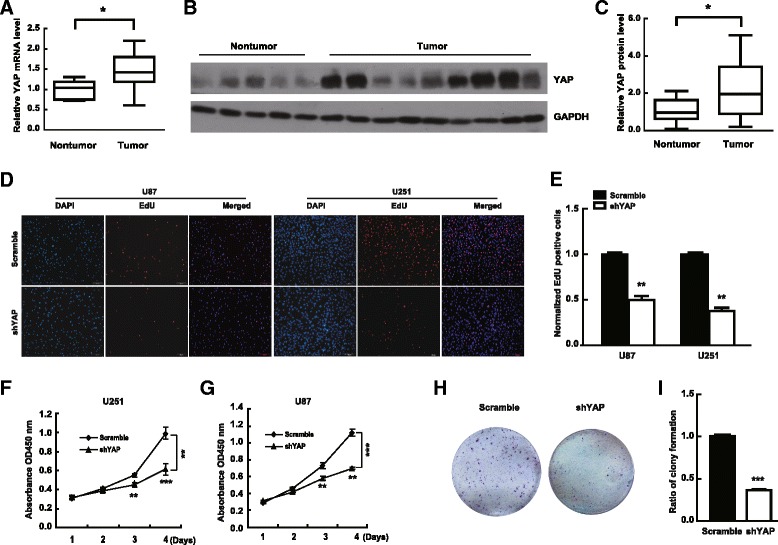
Knockdown of YAP inhibits glioma cell proliferation in vitro*.* **a** qPCR analysis for YAP mRNA levels in glioma (*n* = 18) and nontumor brain tissues (*n* = 6). **b** Representative immunoblot of the total extracts from human gliomas (nine samples shown) and nontumor tissues (five samples shown). **c** Quantification of YAP protein levels in glioma (*n* = 26) and normal tissues (*n* = 13) from the immunoblots. **d** & **e** Representative images of EdU assay after infecting cells with indicated lentivirus (**d**) and quantification results (**e**). The cell proliferation was examined after plating for 48 h. Scale bar, 100 μm. **f** & **g** The effect of YAP knockdown on cell proliferation was detected by CCK-8 assay at the indicated time. **h** & **i** Representative images (**h**) and quantitative analysis (**i**) of U251 colonies infected with indicated lentivirus. Colonies stained with crystal violet 14 days after seeding. * *P* < 0.05; ** *P* < 0.01; *** *P* < 0.001

In order to study the effect of YAP on glioma progression, we firstly examined the mRNA and protein level of YAP in human glioma tissues by real-time PCR and immunoblotting respectively. As shown in Fig. 1a-c, the mRNA and protein levels of YAP in human glioma tissues were significantly higher than that in nontumor tissues, in line with our previous report [8].

Therefore, we down-regulated YAP with shRNA lentivirus to examine the effect of YAP on glioma cell proliferation. As shown in supporting Fig. 1a and b (Additional file 1: Figure S1), the percentage of GFP positive cells (YAP knocking-down cells) was more than 95% and the down-regulation efficiency was about 80% in both U251 and U87 cells. Examined by EdU incoporation assay and CCK-8 assay, depletion of YAP significantly inhibited the proliferation of U251 and U87 cells (Fig. 1d-g). Similarly, the number of the colonies formed from YAP down-regulation cells significantly decreased (Fig. 1h, i). The above results indicate that YAP down-regulation inhibited glioma cell proliferation in vitro.

### Down-regulation of YAP inhibits intracranial glioma growth in vivo

**Fig. 2:**
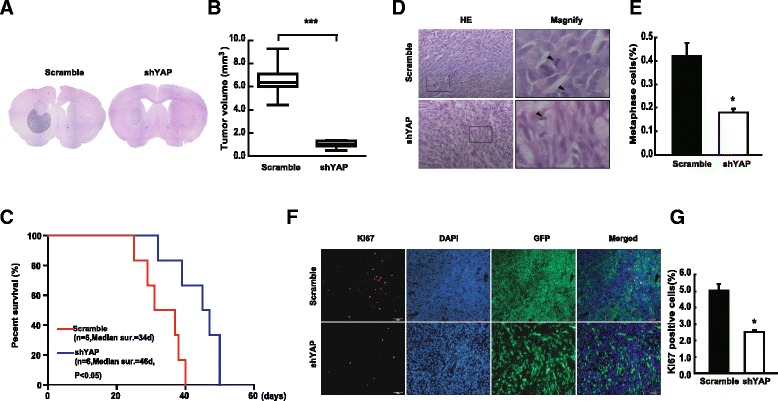
Down-regulation of YAP inhibits intracranial glioma growth in vivo. **a** HE staining of tumors derived from scramble and shYAP cells. **b** Quantitative analysis of the tumor volume. **c** Kaplan-Meier curve of scramble and shYAP mice showed a clear survival advantage for YAP down-regulation (*n* = 6; *P* < 0.05, determined using log-rank test). **d** & **e** The number of metaphase cells was counted per 20 high-power fields (**d**, right: amplified images) and quantified (**e**). Scale bar: 20 μm. **f** & **g** Ki67 positive cells were determined by Ki67 staining. Scale bar: 50 μm. * *P* < 0.05, ** *P* < 0.01

To study the effect of YAP on intracranial glioma growth in vivo, YAP down-regulation U87 cells were transplanted into the right striatum of nude mice. We found that the tumors derived from YAP down-regulation cells were significantly smaller than those of the scramble mice (Fig. 2a, b). In addition, the median survival time was prolonged from 34 days to 46 days after YAP down-regulation (Fig. 2c), indicating that YAP down-regulation mice had a clear survival advantage. The number of mitotic cells and Ki67 positive cells of YAP-down regulation group decreased significantly (Fig. 2d-g). Interestingly, the cell density (GFP positive cells) of tumors derived from YAP down-regulation group was significantly lower than that of scramble group (Fig. 2f). The above results indicated that down-regulation of YAP inhibited intracranial glioma growth in vivo.

### YAP modulates the protein level of β-catenin via regulating the activity of GSK3β in destruction complex

**Fig. 3:**
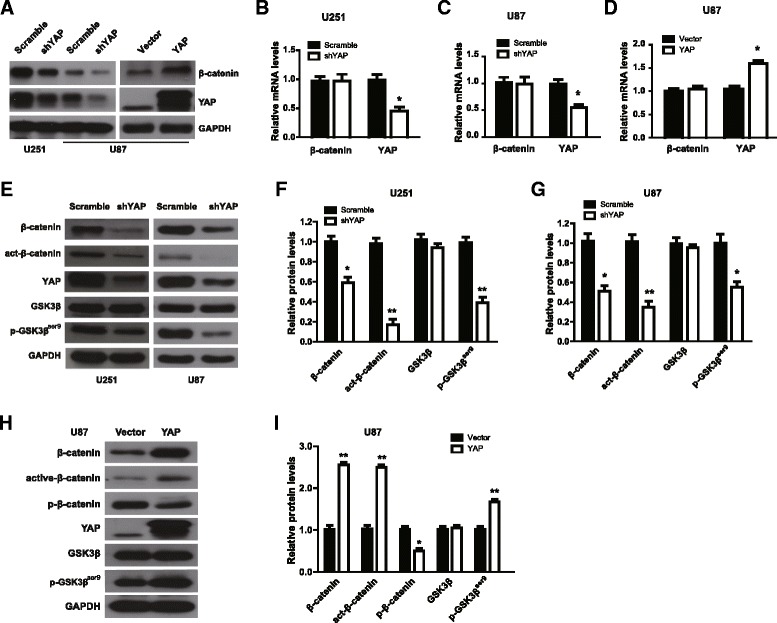
YAP modulates the protein level of β-catenin via regulating the activity of GSK3β in destruction complex. **a** Representative immunoblot of β-catenin in YAP down-regulation or over-expression cells. **b-d** β-catenin mRNA level in YAP down-regulation or over-expression cells was analyzed by qPCR. **e** Representative immunoblot of GSK-3β, p-GSK-3β, β-catenin and active-β-catenin after YAP down-regulation. **f** & **g** Quantification result of (**e**). (**h**) Representative immunoblot of GSK-3β, p-GSK-3β, β-catenin, active-β-catenin and p-β-catenin after YAP over-expression. **i** Quantification result of (**h**). * *P* < 0.05, ** *P* < 0.01

Next, we address how YAP regulates glioma progression. Hippo/YAP pathway is closely related to the Wnt/β-catenin signaling [24, 25]. In addition, we and others have reported that Wnt/β-catenin plays important roles in glioma development [13–15, 26]. Whether YAP regulates β-catenin in human gliomas? We examined the protein and mRNA levels of β-catenin after YAP down-regulation or over-expression. YAP over-expression U87 glioma cells with about 90% GFP-positive percentage were established (Additional file 2: Figure S2A and B). The protein level of β-catenin decreased after YAP down-regulation, while it increased after YAP over-expression (Fig. 3a). However, the mRNA level of β-catenin showed no change (Fig. 3b-d), in line with the previous report [21].

As the protein level of β-catenin is mainly modulated via phosphorylation of GSK3β in AXIN/APC/GSK3β destruction complex [10, 11]**,** we therefore wonder whether YAP modulates the protein level of β-catenin via regulating the activity of GSK3β in destruction complex. As shown in Fig. 3e-g, we found that the protein levels of p-GSK-3β, β-catenin and active-β-catenin decreased when YAP was down-regulated in U87 and U251 cells. On the contrary, the above three proteins increased and p-β-catenin level decreased after YAP over-expression in U87 cells (Fig. 3h, i). Interestingly, YAP S94A, a YAP mutation in the TEAD-binding domain, recapitulated the effect of YAP wild type on GSK3β and β-catenin phosphorylation (Additional file 3: Figure S3A and B), suggesting that YAP regulates GSK3β activity in the cytoplasm and the transcriptional activity of YAP is dispensable for the observed phenotypes.

### YAP modulates the subcellular location and transcription activity of β-catenin

**Fig. 4:**
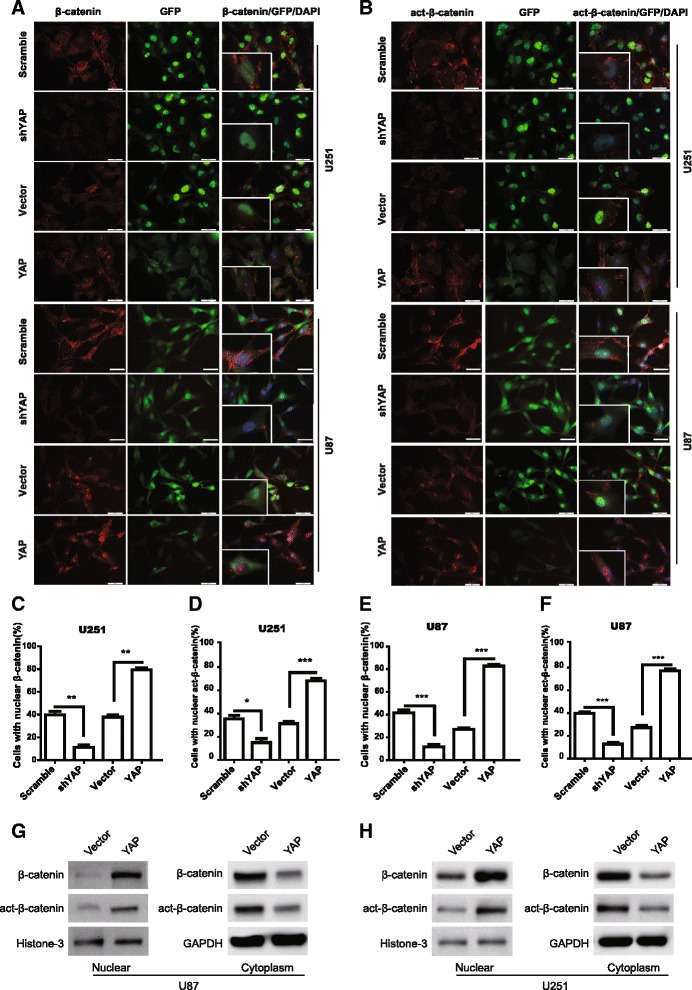
YAP modulates the subcellular location of β-catenin. **a & b** The expression and subcellular location of β-catenin (**a**) and active-β-catenin (**b**) were assessed by immunofluorescence in YAP down-regulation or over-expression cells. Scale bar 50 μm. Inset showed the amplified images. **c-f** Quantification results of the percentage of cells with nuclear β-catenin (**c** & **e**) or active-β-catenin (**d** & **f**) in U251 and U87 cells. * *P* < 0.05, ** *P* < 0.01, *** *P* < 0.001. **g** & **h** Subcellular location of β-catenin or active-β-catenin was detected by using cellular fractionation and immunoblotting. Histone and GAPDH were used as nuclear and cytoplasm loading control respectively

Next, we examined whether YAP affects the subcellular location and activity of β-catenin. As shown in Fig. 4a and b, the fluorescence intensity (the protein level) of β-catenin and active-β-catenin decreased after YAP down-regulation, while it increased after YAP over-expression. Importantly, the subcellular location of the above two proteins was mainly at cytoplasm, cell membrane and the cellular junctions after YAP down-regulation. Interestingly, after YAP over-expression, the above two proteins were mainly located at the nucleus (Fig. 4a and b). The percentage of cells with nuclear β-catenin and active β-catenin increased significantly after YAP over-expression, while it decreased after YAP down-regulation (Fig. 4c-f). In addition, by using cellular fractionation and immunoblotting, we found that the nuclear β-catenin and active β-catenin levels increased after overexpressing YAP in U251 or U87 cells (Fig. 4g and h). The above results indicate that YAP over-expression increases both β-catenin protein level and nucleus translocation.

**Fig. 5:**
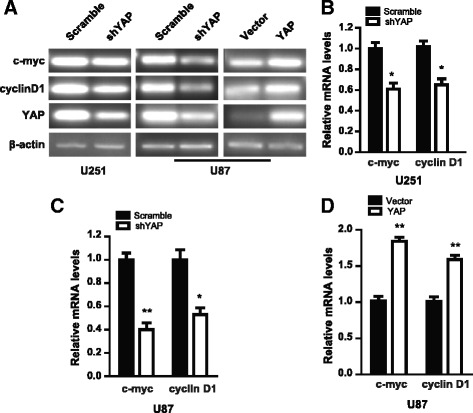
YAP modulates the transcription activity of β-catenin. **a** c-myc and cyclin D1 expression levels were examined in YAP down-regulation or over-expression cells by RT-PCR. **b-d** Quantification result of (**a**). * *P* < 0.05, ** *P* < 0.01

As β-catenin functions as a transcriptional co-activator to promote cell proliferation after being transported into the nucleus [10, 11], we guess that the increased β-catenin and active-β-catenin in the nucleus after YAP over-expression may promote the transcription of β-catenin target genes. As expected, the mRNA levels of c-myc and cyclin D1, two β-catenin target genes, decreased after YAP down-regulation (Fig. 5a-c), while they increased after YAP over-expression (Fig. 5a and d). Together, the above results indicate that YAP enhances the transcription activity of β-catenin in glioma cells.

### The effect of YAP down-regulation on glioma cell proliferation was partially mediated by β-catenin

**Fig. 6:**
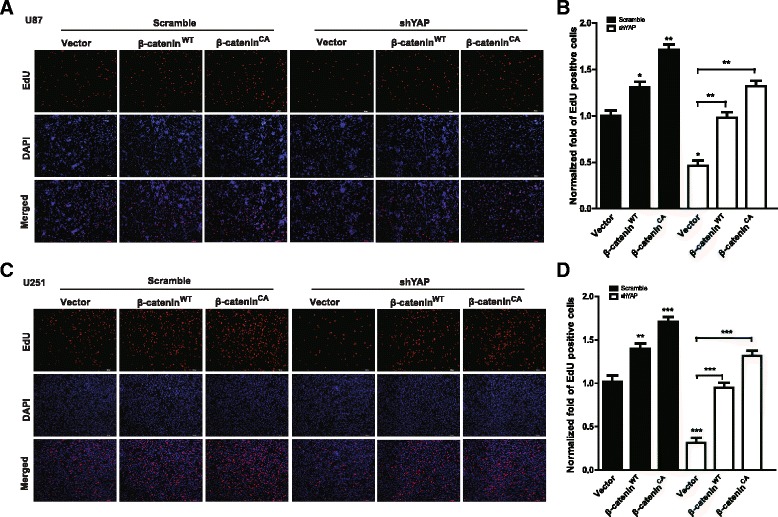
The effect of YAP down-regulation on glioma cell proliferation was partially mediated by β-catenin. **a** & **c** Representative images of EdU assay after over-expression of β-catenin^WT^ and β-catenin^CA^ in U251 (**a**) and U87 (**c**) cells with or without YAP down-regulation. The cell proliferation was examined after plating for 48 h. Scale bar, 200 μm. **b & d** Quantification result of (**a** & **c**). * *P* < 0.05, ** *P* < 0.01

Next, we wonder whether β-catenin mediates the effect of YAP on glioma cell proliferation. We took advantage of β-catenin^WT^ and β-catenin^CA^, the active form of β-catenin, to address this question. β-catenin^CA^ is the mutated form of β-catenin with four serine/threonine residues (Ser33, Ser37, Thr41, and Ser45) changed to alanine, which cannot be phosphorylated by GSK3β and exhibits constitutive stabilization [27, 28]. As shown in Fig. 6a-d, both transient over-expressing β-catenin^WT^ and its active form β-catenin^CA^ in U251 and U87 glioma cells promoted glioma cell proliferation and β-catenin^CA^ exhibited a stronger promotion effect than β-catenin^WT^. In line with our previous results shown in Figs. 1 and 2, down-regulation of YAP inhibited glioma cell proliferation (Fig. 6a-d). Interestingly, when β-catenin^WT^ and β-catenin^CA^ were over-expressed in the cells with YAP depletion, the reduced proliferation capacity of glioma cells induced by YAP down-regulation could be partially rescued. Especially, β-catenin^CA^ exhibited a stronger rescue effect than that of β-catenin^WT^ (Fig. 6a-d). These results demonstrate that YAP increases the protein level and activity of β-catenin via inhibiting the activity of GSK3β, which ultimately promotes the proliferation of glioma cells.

## Discussion

The Hippo/YAP pathway plays a key role in regulating organ size, tissue homeostasis, and patterning [29, 30]. Numerous studies reported that YAP is up-regulated in many types of human cancers and exhibits positive association with patient prognosis [3]. However, YAP also is reported to be down-regulated in some human tumors, such as breast cancer, hematological cancers [31, 32], indicating that YAP may exhibit a tissue- or context-dependent role in tumor biology. In our previous study, we reported that up-regulated YAP1 in human gliomas is positively associated with glioma patient prognosis and transient over-expression of YAP promotes glioma cell proliferation [8]. In the current study, we found that down-regulation of YAP inhibited glioma cell proliferation both in vitro and in vivo, indicating that YAP may be a potential molecular target for glioma treatment.

We found that YAP over-expression increased β-catenin protein level in human gliomas, consistent with the previous report [18]. However, two groups reported that shYAP1 or conditional knockout YAP did not affect β-catenin stability or levels [33, 34]. In our system, we found that neither down-regulation nor over-expression of YAP changed the mRNA level of β-catenin. In line with our finding, Park et al. reported that YAP/TAZ has no effect on the mRNA level of β-catenin and alternatively they are the downstream effectors of Wnt signaling [21]. The difference between above studies may be caused by different experiment system used or different functions of YAP/TAZ in different tissues. For example, the level of YAP decreased in breast cancer and in hematological cancers [31, 32], while it is up-regulated in human gliomas [8]. In addition, YAP, as a component of the β-catenin destruction complex, acts as β-catenin inhibitor in the WNT-OFF state and as Wnt transducer in the WNT-ON state [20]. However, in the current study, we found that YAP over-expression increased β-catenin level, nucleus translocation and transcription activity, indicating that YAP acts as a β-catenin promoter, but not an inhibitor in glioma context.

We found that the protein level of p-GSK-3β increased after YAP over-expression, leading to β-catenin and active-β-catenin increase. In addition, β-catenin^CA^ has a stronger rescue effect than that of β-catenin^WT^ on cell growth inhibition induced by YAP depletion, indicating that YAP inhibits GSK3β activity indeed. How does YAP affect the activity of GSK3β? Xin. et al. reported that, in YAP^S112A^ (a mouse constitutively active form of YAP and localized at the nucleus)-expressing cells, activated YAP enhances IGF signaling by phosphorylating AKT and GSK-3β, leading to GSK-3β activity inhibition. Consistently, the level of β-catenin and the active non-phosphorylated β-catenin increased in YAP^S112A^-expressing cardiomyocytes [35]. In 2014, Azzolin et al. reported that, in Wnt-OFF cells, although down-regulation of YAP does not affect the formation of AXIN/APC/GSK3β complex, it inhibits β-TrCP recruitment, leading to β-catenin activation [20]. In our study, YAP S94A, a human YAP mutation in the TEAD-binding domain, recapitulated the effect of YAP wild type on GSK3β and β-catenin phosphorylation (Additional file 1: Figure S3A and B), suggesting that YAP regulates GSK3β activity in the cytoplasm and the transcriptional activity of YAP is dispensable for the observed phenotypes.

We observed that, after YAP over-expression, the increased β-catenin and active-β-catenin translocated into the nucleus, leading to c-myc and cyclin D1 transcription. As nuclear accumulation of β-catenin participates in malignant progression of gliomas and implicates poor prognosis [36], we deduce that the nucleus increased β-catenin and active-β-catenin after YAP over-expression may be related to the incidence and development of gliomas. Interestingly, over-expressed YAP (GFP-YAP) is mainly concentrated in the cytoplasm in the glioma cells (Fig. 4a, Additional file 2: Figure S2A) and β-catenin was mainly located in cytomembrane and the cellular junctions (Fig. 4a and b), how does over-expression of YAP promote β-catenin into the nucleus? Imajo. et al. demonstrated that phosphorylated YAP suppresses nuclear translocation of β-catenin by directly binding to it in the cytoplasm [18]. In Wnt-ON cells, release of YAP from the AXIN/APC/GSK3β complex promotes both YAP and β-catenin into nucleus [20]. However, both studies cannot explain our findings, in which YAP is accumulated in the cytoplasm and β-catenin in the nucleus. Whether over-expressed YAP competes with β-catenin for binding AXIN/APC/GSK3β complex and then releases β-catenin from the complex in glioma context? The mechanism that β-catenin translocation between the nucleus and cytoplasm after YAP over-expression in glioma cells need to be explored in the future.

## Conclusion

We found that knockdown of YAP inhibited glioma cell proliferation in vitro and tumor growth in vivo. Our results identify a novel mechanism that YAP modulates the protein level, subcellular location and transcription activity of β-catenin via regulating the activity of GSK3β in destruction complex. β-catenin mediates the promoting effect of YAP on glioma cell proliferation, which provides a new crosstalk mechanism between Hippo/YAP and Wnt/β-catenin pathways and suggests a new strategy for human gliomas treatment.

### Ethics approval and consent to participate

The use of human tissues was approved by the Ethics Committee of the Affiliated Hospital of Xuzhou Medical University (No: xyfylw2014002). Written informed consent was obtained from each patients. All animal experiments were performed according to the guidelines for the care and use of laboratory animals and were approved by IACUC of Xuzhou Medical University (No: 201,647).

## Additional files

Additional file 1: Figure S1. Generation of YAP down-regulation U87 and U251 cells. **(A)** The infection efficiency of scramble and shYAP lentivirus in U251 and U87 cells. U87 and U251 glioma cells were infected with viral supernatant and the infection efficacy was evaluated by GFP positive cells 72 h later. Scale bar, 100 μm. PH: Phase contrast. **(B)** Representative immunoblots of protein extraction from shYAP infected U251 and U87 glioma cells (up panel)and YAP protein levels were quantified (bottom panel). *** *P* < 0.001. (PDF 358 kb)

Additional file 2: Figure S2. Generation of YAP over-expression U87 cells. **(A)** The infection efficiency of vector and YAP lentivirus in U87 cells.U87 glioma cells were infected with viral supernatant and the infection efficacy was evaluated by GFP positive cells 72 h later.Scale bar, 100 μm. PH: Phase contrast. **(B)** Expression analysis of YAP protein levels by Western blotting in YAP over-expression U87 glioma cells. (PDF 90 kb)

Additional file 3: Figure S3. The effect of YAP on GSK-3β and β-catenin phosphorylation. **(A&B)** Representative immunoblots of GSK-3β, p-GSK-3β, β-catenin, active-β-catenin and p-β-catenin after over-expression of YAP or YAP S94A in U251 **(A)** and U87 **(B)** glioma cells.The results indicate that YAP S94A recapitulated the effect of YAP wild type on GSK3β and β-catenin phosphorylation. (PDF 153 kb)
