# Correction: β-catenin-mediated YAP signaling promotes human glioma growth

**DOI:** 10.1186/s13046-025-03415-6

**Published:** 2025-05-31

**Authors:** Yan Wang, Peng Pan, Zhaohao Wang, Yu Zhang, Peng Xie, Decheng Geng, Yang Jiang, Rutong Yu, Xiuping Zhou

**Affiliations:** 1 Insititute of Nervous System Diseases, Xuzhou Medical University, 84 West Huai-Hai Road, Xuzhou, Jiangsu 221002 People’s Republic of China; 2 Brain Hospital, Affiliated Hospital of Xuzhou Medical University, Xuzhou, Jiangsu China; 3 The Graduate School, Xuzhou Medical University, Xuzhou, Jiangsu China; 4 Present Address: Department of Neurosurgery, Xuzhou Cancer Hospital, Xuzhou, Jiangsu China; 5 Jiangsu Center for the Collaboration and Innovation of Cancer Biotherapy, Cancer Institute, Xuzhou Medical University, Xuzhou, Jiangsu China

**Correction: J Exp Clin Cancer Res 36, 136 (2017)**

**https://doi.org/10.1186/s13046-017–0606-1**

Following the publication of the original article [1], the authors found errors in the figures, specifically, Fig. 4a and Fig. 6c where the IF image of Scramble group in U251 cell and the images of β-catenin(WT) and β-catenin(CA) in shYAP group were incorrect.

Below are the correct figures:

**Incorrect Figure 4**

**Fig. 4:**
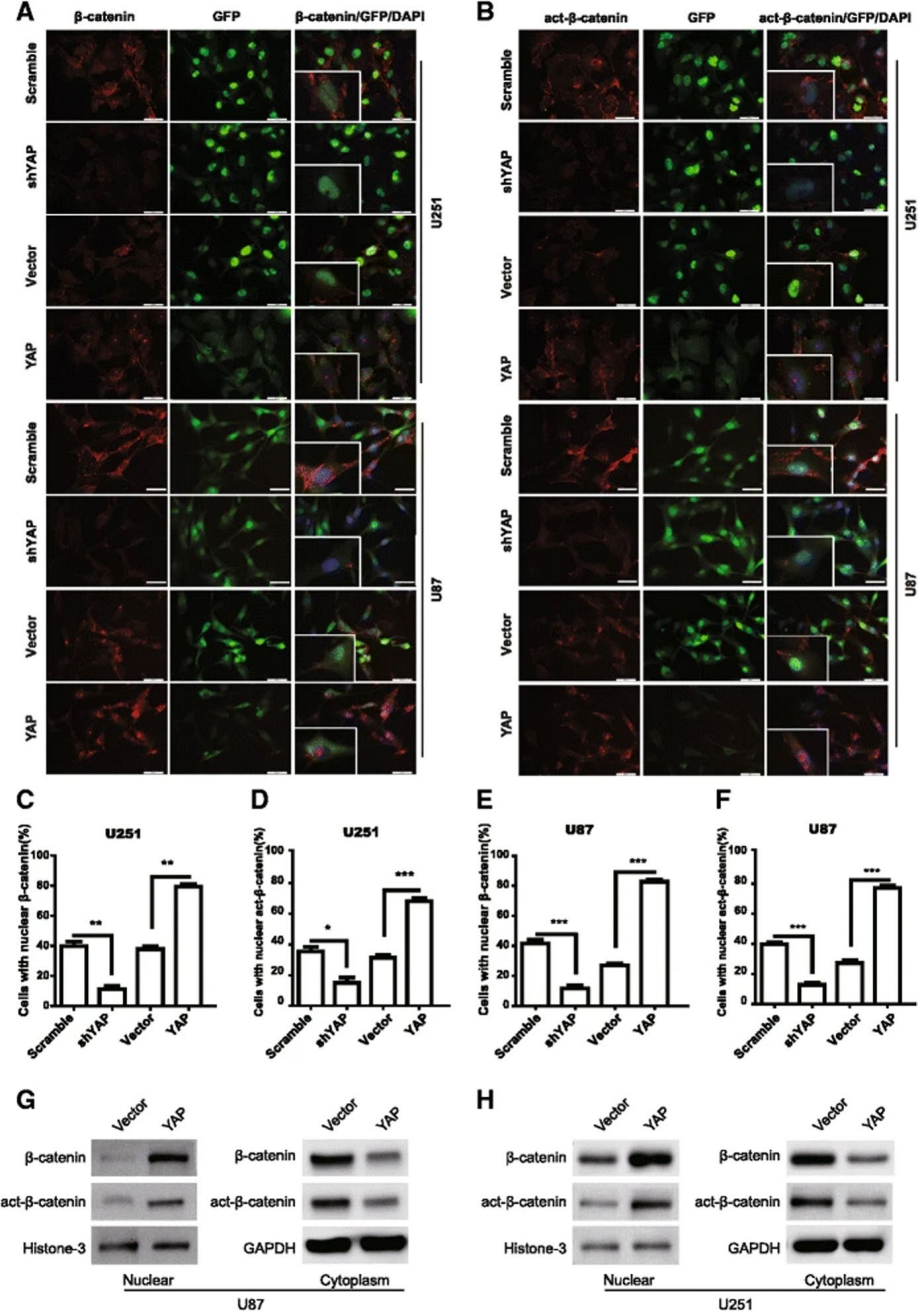
YAP modulates the subcellular location of β-catenin. **a**&**b** The expression and subcellular location of β-catenin (**a**) and active-β-catenin (**b**) were assessed by immunofluorescence in YAP down-regulation or over-expression cells. Scale bar 50 μm. Inset showed the amplified images. **c-f** Quantification results of the percentage of cells with nuclear β-catenin (**c** & **e**) or active-β-catenin (**d** & **f**) in U251 and U87 cells. * *P* < 0.05, ** *P* < 0.01, *** *P* < 0.001. **g **&** h** Subcellular location of β-catenin or active-β-catenin was detected by using cellular fractionation and immunoblotting. Histone and GAPDH were used as nuclear and cytoplasm loading control respectively

**Correct Figure 4**

**Fig. 4:**
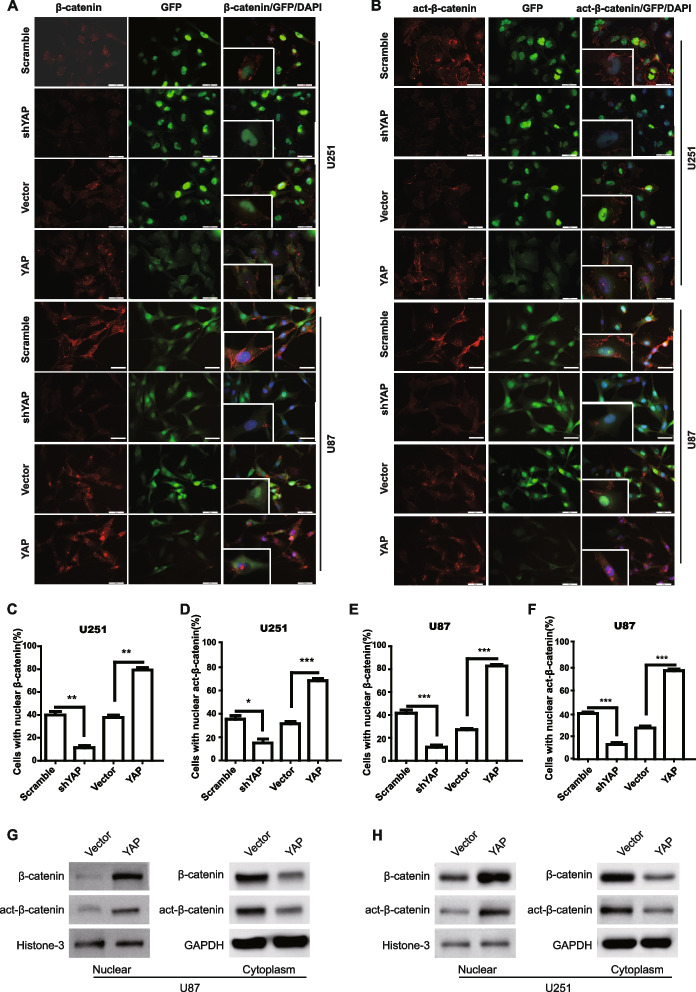
YAP modulates the subcellular location of β-catenin. **a**&**b** The expression and subcellular location of β-catenin (**a**) and active-β-catenin (**b**) were assessed by immunofluorescence in YAP down-regulation or over-expression cells. Scale bar 50 μm. Inset showed the amplified images. **c-f** Quantification results of the percentage of cells with nuclear β-catenin (**c** & **e**) or active-β-catenin (**d** & **f**) in U251 and U87 cells. * *P* < 0.05, ** *P* < 0.01, *** *P* < 0.001. **g **&** h** Subcellular location of β-catenin or active-β-catenin was detected by using cellular fractionation and immunoblotting. Histone and GAPDH were used as nuclear and cytoplasm loading control respectively

**Incorrect Figure 6**

**Fig. 6:**
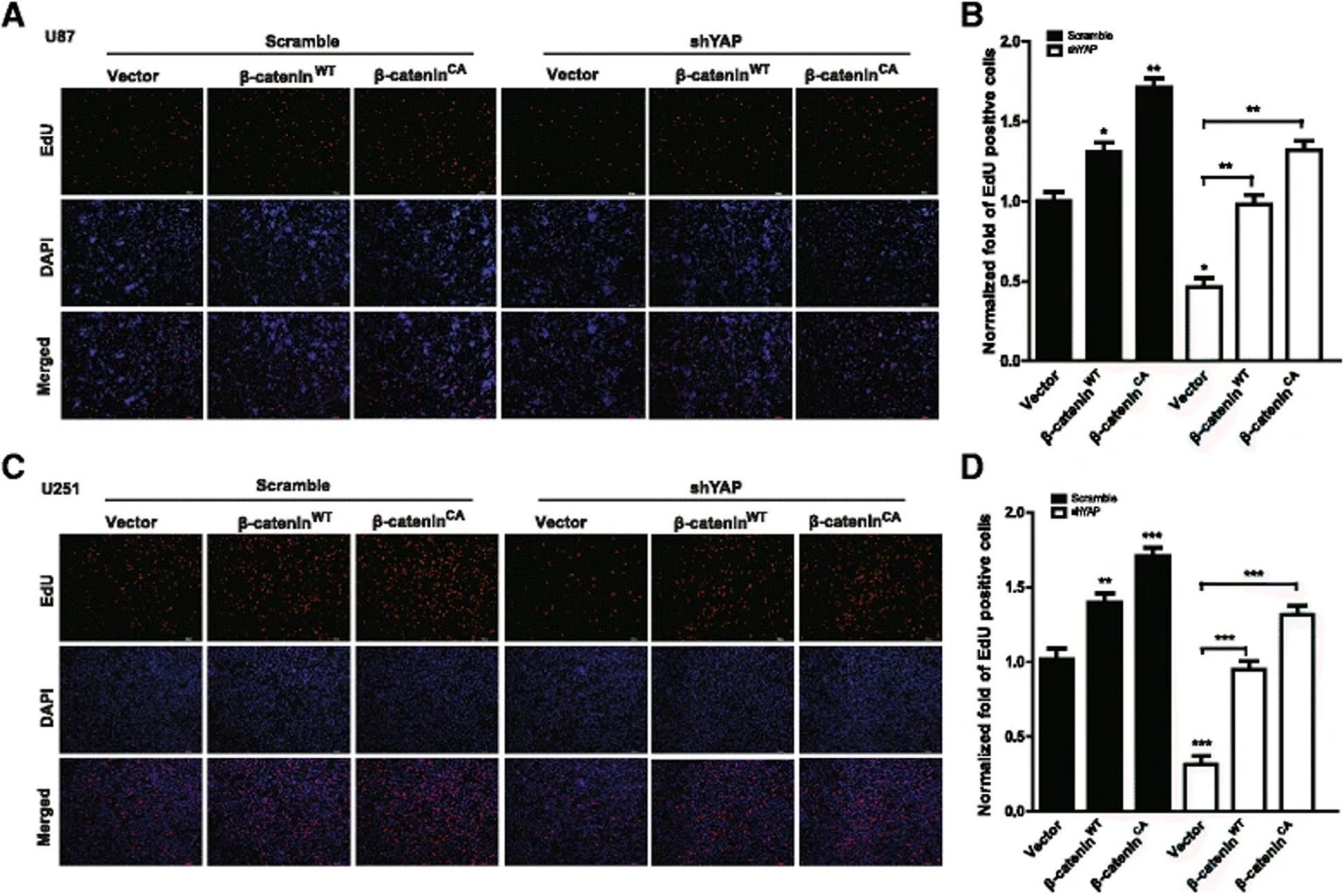
The effect of YAP down-regulation on glioma cell proliferation was partially mediated by β-catenin. **a** & **c** Representative images of EdU assay after over-expression of β-catenin^WT^ and β-catenin^CA^ in U251 (**a**) and U87 (**c**) cells with or without YAP down-regulation. The cell proliferation was examined after plating for 48 h. Scale bar, 200 μm. **b**&**d** Quantification result of (**a** & **c**). * *P* < 0.05, ** *P* < 0.01

**Correct Figure 6**

**Fig. 6:**
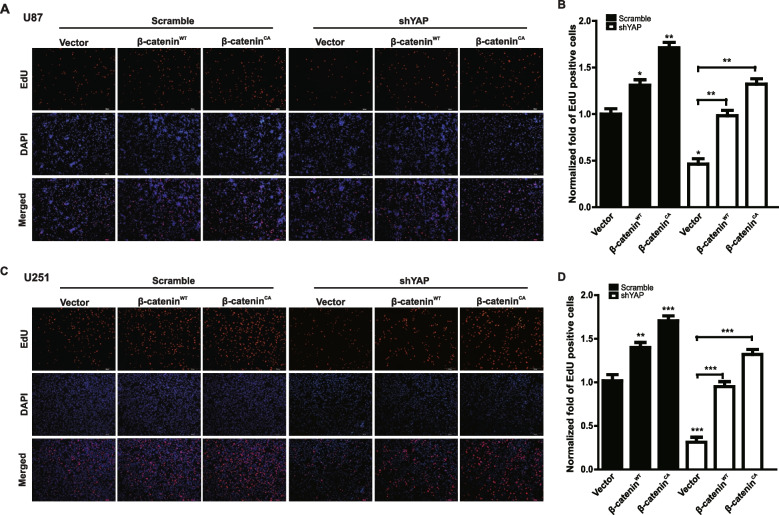
The effect of YAP down-regulation on glioma cell proliferation was partially mediated by β-catenin. **a** & **c** Representative images of EdU assay after over-expression of β-catenin^WT^ and β-catenin^CA^ in U251 (**a**) and U87 (**c**) cells with or without YAP down-regulation. The cell proliferation was examined after plating for 48 h. Scale bar, 200 μm. **b**&**d** Quantification result of (**a** & **c**). * *P* < 0.05, ** *P* < 0.01

The corrections do not compromise the validity of the conclusions and the overall content of the article. The original article [1] has been updated.
