# Supplementary material for: β-catenin-mediated YAP signaling promotes human glioma growth
Source: J Exp Clin Cancer Res. 2017 Sep 29;36:136. doi: 10.1186/s13046-017-0606-1 (PMC5622484; doi:10.1186/s13046-017-0606-1)
Supplement: Supplementary file 1 — Generation of YAP down-regulation U87 and U251 cells. (A) The infection efficiency of scramble and shYAP lentivirus in U251 and U87 cells. U87 and U251 glioma cells were infected with viral supernatant and the infection efficacy was evaluated by GFP positive cells 72 h later. Scale bar, 100 μm. PH: Phase contrast. (B) Representative immunoblots of protein extraction from shYAP infected U251 and U87 glioma cells (up panel)and YAP protein levels were quantified (bottom panel). *** P < 0.001. (PDF 358 kb) [file 13046_2017_606_MOESM1_ESM.pdf]

Additional file 1

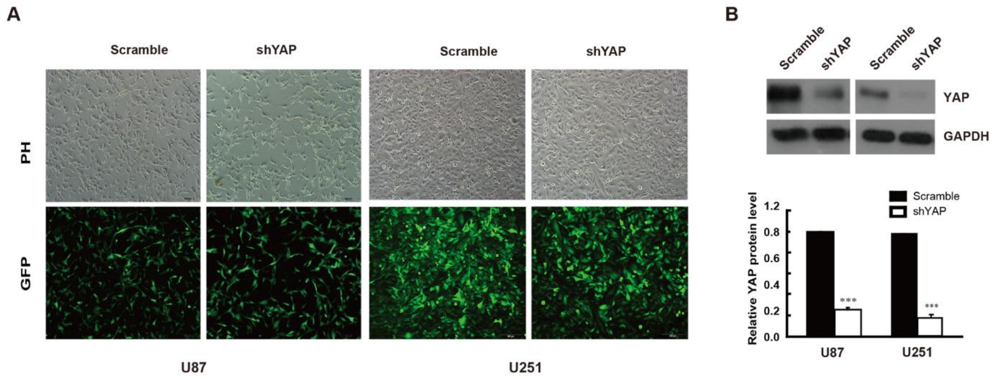

sFig.1 Generation of YAP down-regulation U87 and U251 cells. (A) The infection efficiency of scramble and shYAP lentivirus in U251 and U87 cells. U87 and U251 glioma cells were infected with viral supernatant and the infection efficacy was evaluated by GFP positive cells 72 hours later. Scale bar, 100 µm. PH: Phase contrast. **(B)** Representative immunoblots of protein extraction from shYAP infected U251 and U87 glioma cells (up panel) and YAP protein levels were quantified (bottom panel). *** *P*<0.001.
