# Supplementary material for: β-catenin-mediated YAP signaling promotes human glioma growth
Source: J Exp Clin Cancer Res. 2017 Sep 29;36:136. doi: 10.1186/s13046-017-0606-1 (PMC5622484; doi:10.1186/s13046-017-0606-1)
Supplement: Supplementary file 2 — Generation of YAP over-expression U87 cells. (A) The infection efficiency of vector and YAP lentivirus in U87 cells.U87 glioma cells were infected with viral supernatant and the infection efficacy was evaluated by GFP positive cells 72 h later.Scale bar, 100 μm. PH: Phase contrast. (B) Expression analysis of YAP protein levels by Western blotting in YAP over-expression U87 glioma cells. (PDF 90 kb) [file 13046_2017_606_MOESM2_ESM.pdf]

Additional file 2

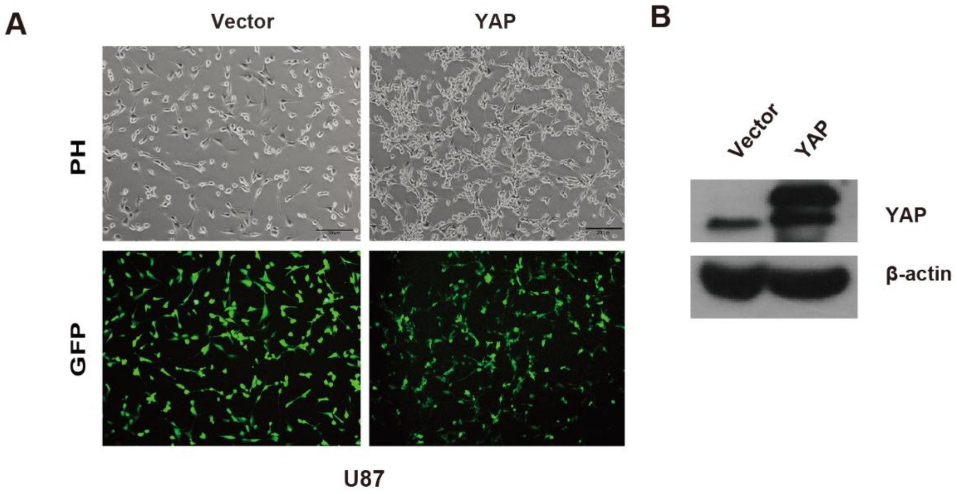

sFig.2 Generation of YAP over-expression U87 cells. **(A)** The infection efficiency of vector and YAP lentivirus in U87 cells. U87 glioma cells were infected with viral supernatant and the infection efficacy was evaluated by GFP positive cells 72 hours later. Scale bar, 100 μm. PH: Phase contrast. **(B)** Expression analysis of YAP protein levels by Western blotting in YAP over-expression U87 glioma cells.
