# Supplementary material for: β-catenin-mediated YAP signaling promotes human glioma growth
Source: J Exp Clin Cancer Res. 2017 Sep 29;36:136. doi: 10.1186/s13046-017-0606-1 (PMC5622484; doi:10.1186/s13046-017-0606-1)
Supplement: Supplementary file 3 — The effect of YAP on GSK-3β and β-catenin phosphorylation. (A&B) Representative immunoblots of GSK-3β, p-GSK-3β, β-catenin, active-β-catenin and p-β-catenin after over-expression of YAP or YAP S94A in U251 (A) and U87 (B) glioma cells.The results indicate that YAP S94A recapitulated the effect of YAP wild type on GSK3β and β-catenin phosphorylation. (PDF 153 kb) [file 13046_2017_606_MOESM3_ESM.pdf]

Additional file 3

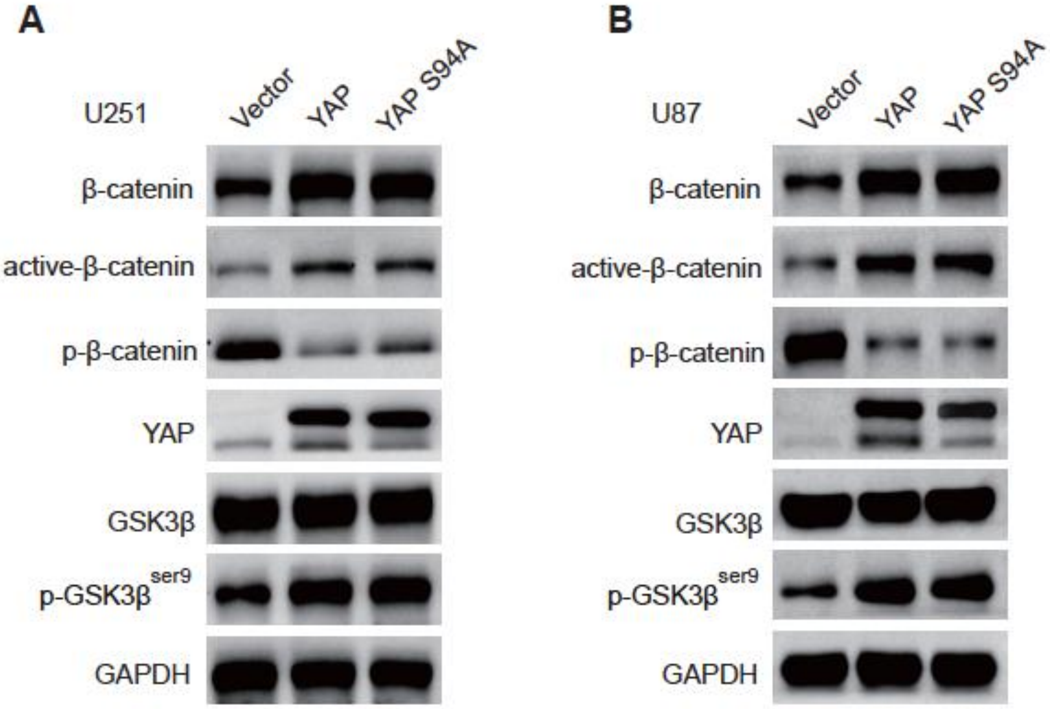

sFig.3 The effect of YAP on GSK-3β and β-catenin phosphorylation. (A&B) Representative immunoblots of GSK-3β, p-GSK-3β, β-catenin, active-β-catenin and p-β-catenin after over-expression of YAP or YAP S94A in U251 **(A)** and U87 **(B)** glioma cells. The results indicate that YAP S94A recapitulated the effect of YAP wild type on GSK3β and β-catenin phosphorylation
